# Learning the Macroscopic Flow Model of Short Fiber Suspensions from Fine-Scale Simulated Data

**DOI:** 10.3390/e22010030

**Published:** 2019-12-24

**Authors:** Minyoung Yun, Clara Argerich Martin, Pierre Giormini, Francisco Chinesta, Suresh Advani

**Affiliations:** 1 Processes and Engineering in Mechanics and Materials (PIMM) Laboratory, Arts et Métiers Institute of Technology, CNRS, CNAM, 151 Boulevard de l’Hôpital, 75013 Paris, France; minyoung.yun@ensam.eu (M.Y.); clara.argerich_martin@ensam.eu (C.A.M.); Pierre.GILORMINI@ensam.eu (P.G.); 2 ESI Group chair, Processes and Engineering in Mechanics and Materials (PIMM) Laboratory, Arts et Métiers Institute of Technology, 151 Boulevard de l’Hôpital, 75013 Paris, France; 3 Center for Composite Materials and Department of Mechanical Engineering, University of Delaware, Newark, DE 19716-3119, USA; advani@udel.edu

**Keywords:** fiber suspensions, data-driven modeling, machine learning

## Abstract

Fiber–fiber interaction plays an important role in the evolution of fiber orientation in semi-concentrated suspensions. Flow induced orientation in short-fiber reinforced composites determines the anisotropic properties of manufactured parts and consequently their performances. In the case of dilute suspensions, the orientation evolution can be accurately described by using the Jeffery model; however, as soon as the fiber concentration increases, fiber–fiber interactions cannot be ignored anymore and the final orientation state strongly depends on the modeling of those interactions. First modeling frameworks described these interactions from a diffusion mechanism; however, it was necessary to consider richer descriptions (anisotropic diffusion, etc.) to address experimental observations. Even if different proposals were considered, none of them seem general and accurate enough. In this paper we do not address a new proposal of a fiber interaction model, but a data-driven methodology able to enrich existing models from data, that in our case comes from a direct numerical simulation of well resolved microscopic physics.

## 1. Introduction

Short fiber reinforced composite is being used in a wide range of sectors in our society. The composite is manufactured from processes such as, among many others, injection molding, compression molding and extrusion. The mechanical performances of the manufactured composite, such as stiffness and strength, is determined by the final state of fiber orientation. The final fiber state results from the flow induced orientation evolution that in the case of semi-concentrated or concentrated flow regimes involves strong fiber–fiber interactions whose modeling is crucial for determining the final part properties and performance.

Predicting flow induced anisotropy in reinforced polymer forming processes is of major interest. However, most of the existing models, as the ones described below, have important limitations associated with the numerous hypotheses introduced during their analytical derivation. When proceeding out of the domain of validity of those models, a pragmatic approach consists of assuming the existing models are still valid and calibrating them from experimental tests.

When these calibrated nominal models are unable to reproduce experimental findings, some ad hoc terms are usually added, being then calibrated accordingly (e.g., diffusion term reflecting fiber interactions).

Thus, data was traditionally employed for calibrating models derived from physical considerations. However, many times those models failed to address experimental findings even when they were finely calibrated. It is the case of models describing intense fiber interactions in semi-concentrated and concentrated fiber suspensions. This issue motivated numerous works referred to later, and remains even today not totally solved.

Recent advances in artificial intelligence, and more particularly in machine learning, are opening new routes. Thus, data can be used not only for calibrating pre-assumed models, i.e., assimilating data into existing models to identify the most adequate values of their parameters. Data can be also used for inferring the model itself, or eventually the correction of a nominal pre-existing model to enhance its predictability capacity.

The present paper aims at addressing this important and timely topic of data-driven model enrichment, here addressed from a methodological viewpoint. Future works will focus on (i) the use of data-driven models for improving computational efficiency in the simulation of complex flows as the ones encountered in processing; (ii) the derivation of more accurate rheological models for describing complex fluids; and (iii) the use of these more accurate rheological models in processing simulations for circumventing, or at least alleviating, the limitation of current models and procedures and their associated predictions.

### Revisiting Standard Flow-Induced Orientation Models

The orientation description and its flow induced evolution can be addressed at three different scales [1]:

- At the microscopic scale the orientation state is described from the orientation of each fiber. Thus, in the case of a population consisting of N fibers of length 2L with an almost infinite aspect ratio (length to diameter), all of them are located into a representative volume element –RVE– ω, the orientation state in ω at time *t* reduces to the knowledge of the N unit vectors $p_{i}\left(t\right)$, centered at the *i*-fiber centre of gravity $G_{i}$ and aligned along its axis.
  If we assume that population immersed in a fluid flow characterized by the velocity gradient ∇v, assumed unperturbed by the fibers’ presence and orientation, when neglecting the fiber–fiber interactions, the fiber rotary velocity is expressed by the Jeffery equation [2], which reads
  (1) $${\dot{p}}_{i}=\nabla vp_{i}-\left(p_{i}^{T}\nabla vp_{i}\right)p_{i},\;\;\;\forall i=1,\cdots ,N.$$
- The mesoscopic description makes use of the probability distribution function Ψ(x,t,p), giving the fraction of fibers that at position x and time *t* are oriented along p.
  Again, when neglecting fiber interaction, the evolution of that distribution function is given by the Fokker–Planck equation
  (2) $$\dot{\Psi }+\nabla_{p}\left(\dot{p}\Psi \right)=0,$$
  where $\dot{\Psi }$ represents the material derivative and $\nabla_{p}\left(•\right)$ the gradient operator involving the conformational coordinates p.
- The main difficulty of the previous descriptions is their complexity, the microscopic one due to the fact of needing to track all the fibers immersed in the flow and the mesoscopic due to the fact that the distribution function is defined in a high-dimensional space involving the physical space x, the time *t* and the orientation p, the last defined on the unit sphere. Macroscopic descriptions circumvent both difficulties by operating with the probability distribution function moments [3]. Thus, the second moment of the probability distribution function, also known as second order orientation tensor a(x,t), reads
  (3) a=∮p⊗pΨdp,
  where the integral applies on the unit sphere surface and “⊗” denotes the tensor product.
  As described in [1], the second order moment time evolution, again ignoring fiber interaction, reads
  (4) $$\dot{a}=\nabla va+a{\left(\nabla v\right)}^{T}-2A:\nabla v,$$
  where A is the fourth order moment and “:” represents the tensor product twice contracted. In what follows the orientation rate in Equation (4) will be referred to as ${\dot{a}}^{Jeff}$ for indicating that it is associated to the Jeffery model that ignores fiber–fiber interactions.

The macroscopic description is retained in most of works and commercial software because of its relative simplicity. However, for employing it, one must consider closure relations [4] for expressing the fourth order orientation tensor as a function of the second one, whose validity remains nowadays an open issue. In the numerical tests performed in the present paper the IBOF closure [5] (invariant-based orthotropic fitted) is considered. Moreover, by restricting the orientation description to the second moment, only some probability distribution functions can be accurately described, others cannot be expressed from the solely use of the second order orientation tensor. On the other hand, size effects were neglected when deriving the models at different scales, limiting the model validity when addressing confined flows.

In the discussion above, fiber–fiber interaction was ignored. Pioneering works assumed that interactions can be considered as randomizing events that tends to push the systems towards an isotropic orientation state, and then, they were modeled from a diffusion term into the Fokker-Planck equation by Folgar and Tucker [6]. Thus, the probability distribution function time evolution is now expressed from

(5) $$\dot{\Psi }+\nabla_{p}\left(\dot{p}\Psi \right)=\nabla_{p}\left(D\nabla_{p}\Psi \right),$$

with *D* the diffusion coefficient, assumed scaling the flow intensity from the second invariant of the velocity gradient.

By using this expression it results in an orientation evolution equation composed of two terms, the usual Jeffery-based orientation model complemented with a correction that can be easily derived as illustrated in [1]. Thus the time evolution of the orientation can be written in its most general form as

(6) $$\dot{a}={\dot{a}}^{Jeff}+{\dot{a}}^{dev},$$

where the first term corresponds to the Jeffery contribution given by Equation (4) and the second contribution, the deviation with respect to the Jeffery model, comes from the interactions, modeled as a diffusion term within the Folgar and Tucker (F&T) model.

For addressing anomalous experimental orientation findings at odds with F&T predictions, in particular a delay in the orientation rates, other models of fiber–fiber interactions have been proposed [7,8,9,10,11,12]. In these studies, the observed delay was attributed to the intense fiber–fiber interactions and modeled either with a modified diffusion term, a microscopic description of interactions, or by introducing a sliding mechanism between fibers and fluid, with an anisotropic rotary diffusion.

At present neither, closure relations nor interaction models that are general and accurate enough, exist. A recent comparison of different approaches can be found in [13]. The accuracy of the fiber orientation is tested usually by predicting the effect on mechanical properties due to the error in predicting of fiber orientation. This issue is beyond the scope of this work but references [3,4] have done that comparison. In [14] authors noticed that for example, the incorrect prediction of fiber orientation as the suspension flows into a rib could seriously compromise the part performance.

Despite the fact that numerous works continue to introduce improved models, the present paper proposes an alternative methodology for improving existing models, based on the data-driven enriching of the model.

In our former works [15] we addressed the fully data-driven modeling of suspensions, here we restrict to enriching the Jeffery model with a data-driven description of interactions, a methodology also considered in some of our former works [16,17].

As our main aim is not the derivation of a new interaction model for fiber suspensions, in what follows we will consider a molecular dynamics (MD) simulation, able to represent in absence of interactions Jeffery motion, and in presence of interactions describing them with a a standard Lennard–Jones potential. Here we are only interested in assimilating that data extracted from the data-driven interaction model that when added to the Jeffery contribution enables an accurate prediction of the orientation evolution in flow conditions that are different from the ones used to extract the model.

This fine-scale simulated data-generator does not pretend to infer an improved model for simulating processes, but only proving that a macroscopic orientation model can be learned from microscopic data in order to accurately describe that orientation state. By having access to more accurate data associated with the orientation evolution of a population of rods, from adequate measurements or generated from more accurate microscopic models (direct numerical simulations solving the fluid kinematics and induced fibers motion) more accurate macroscopic models could be derived.

The methodology described in this paper remains totally general, the only difference being the quality of the data that serves for learning the model, and consequently it can be applied to any flow regime, from dilute (without major interest being the Jeffery model valid) to the highly concentrated regime (where standard model fail).

The next section addresses the data-generator based on a MD fine-scale simulation. Then in Section 3 we introduce the machine learning techniques that will be applied in Section 4 to extract the interaction term. Finally in Section 5 we proceed to verify the proposal by conducting some numerical experiments.

## 2. Data Generator Based on Molecular Dynamics (MD) Simulations

The fine scale simulation is performed by representing the fiber as M connected beads where forces apply, the last derived from different potentials as well as the hydrodynamic drag.

We consider three different potentials: The Lennard–Jones potential $V^{LJ}$ to describe bead-bead interaction (both beads belonging to different fibers) and $V^{E}$ and $V^{B}$ that describe the elongation and bending interaction between neighbor beads of the same fiber.

As indicated at the end of the previous section, the MD simulation here addressed allows recovering Jeffery model predictions in absence of interaction. When interactions occur nothing was demonstrated on the ability of MD for representing fibers motion, but here as previously indicated, our main aim is proving that a macroscopic model can be extracted from individual microscopic data. The quality of the learned model only depends on the quality of the data that served to extract it. The MD strategy here considered must be viewed as a simply synthetic data generator.

Thus, without loss of generality, in what follows we consider the following potential for addressing the just mentioned mechanisms

(7) $$V=\sum_{j=1}^{N}\sum_{i=1}^{M-1}V_{i,i+1;j}^{E}+\sum_{j=1}^{N}\sum_{i=2}^{M-1}V_{i-1,i,i+1;j}^{B}+\sum_{j=1}^{N}\sum_{l=1}^{N}\sum_{i=1}^{M}\sum_{k=1}^{M}V_{i,j;k,l}^{LJ}\chi_{j,l}$$

where N is the number of fibers in the population and $V_{i,j;k,l}^{LJ}$ represents the Lennard–Jones potential between bead *i* of fiber *j* and bead *k* of fiber *l*. The characteristic function $\chi_{j,l}=1-\delta_{jl}$ (with δ the Kroenecker delta) ensures that the Lennard–Jones potential only acts between beads belonging to different fibers.

The Lennard–Jones potential reads

(8) $$V_{i,j;k,l}^{LJ}=4\varepsilon \left({\left(\frac{\sigma }{d_{ik}}\right)}^{12}-{\left(\frac{\sigma }{d_{ik}}\right)}^{6}\right),$$

with $d_{ik}=\left|\right|x_{k}-x_{i}\left|\right|$ the distance between beads $B_{i}$ of fiber *j* and bead $B_{k}$ of fiber *l*, located at positions $x_{i}$ and $x_{k}$ respectively, and ϵ and σ are the two usual parameters involved in the Lennard–Jones potential. The elongation potential is given by

(9) $$V_{i,i+1}^{E}=\frac{K_{E}}{2}{\left(1-\frac{d_{ij}}{d_{eq}}\right)}^{2},$$

where $d_{eq}$ is the equilibrium distance between two successive beads, distance at which the potential reaches its minimum (and consequently the associated force vanishes). In the previous expression $K_{E}$ reflects the potential intensity.

The bending potential between three successive beads

(10) $$V_{i-1,i,i+1}^{B}=\frac{K_{B}}{2}{\left(\theta_{i-1,i,i+1}-\theta_{eq}\right)}^{2},$$

where $\theta_{i-1,i,i+1}$ is the angle defined by vectors joining beads i−1,i and i,i+1 with $\theta_{eq}$ the angle at equilibrium, that vanishes when considering straight fibers. In our study we consider parameters ensuring an almost rigid behavior of the fiber.

The hydrodynamic drag was enforced by adding at each bead the external force

(11) $$F_{i}^{D}=\xi \left(v\left(x_{i}\right)-{\dot{x}}_{i}\right),$$

where ${\dot{x}}_{i}$ denotes the velocity of bead $B_{i}$ and ξ the friction coefficient.

The total force applied on each bead, inserted into the Newton equation, allows the acceleration calculation, and from it the velocity and position update, as performed in standard MD simulations.

The problem is expressed in a dimensionless form prior to proceed with its solution. It is important to emphasize that in absence of Lennard–Jones interactions and for almost rigid fibers, the previous modeling framework recovers the usual Jeffery kinematics.

## 3. Methods

Two dimensionality reduction methods, the principal component analysis (PCA) and the *Code2Vect* will be employed for constructing the interaction data-driven model, the former for reducing the dimensionality and the last for creating the nonlinear regression. For the sake of completeness both are summarized in the next sections.

### 3.1. Principal Component Analysis (PCA)

Let us consider a vector $y\in R^{D}$ containing some data. If some correlations exists in that data, one could find a linear transformation W to transform y into x, with $x\in R^{d}$, with d<D. This mapping reads

(12) y=Wx.

The transformation matrix W, D×d, satisfies the orthogonality condition $W^{T}W=I_{d}$, where $I_{d}$ represents the d×d-identity matrix ($WW^{T}$ is not necessarily $I_{D}$).

Assume that there exist *M* different snapshots $y_{1},\ldots ,y_{M}$, which we store in the columns of a D×M matrix Y. The associated d×M reduced matrix X contains the associated vectors $x_{i}$, i=1,…,M. We assume without loss of generality centered variables.

PCA proceeds by guaranteeing maximal preserved variance and enforcing the latent variables in x being uncorrelated. In other words, the covariance

(13) $$C_{xx}=E\left\{XX^{T}\right\},$$

will be diagonal. PCA extracts the *d* uncorrelated latent variables from

(14) $$C_{yy}=E\left\{YY^{T}\right\}=E\left\{WXX^{T}W^{T}\right\}=WE\left\{XX^{T}\right\}W^{T}=WC_{xx}W^{T}.$$

Pre- and post-multiplying by $W^{T}$ and W, respectively, and making use of the fact that $W^{T}W=I_{d}$, gives us

(15) $$C_{xx}=W^{T}C_{yy}W.$$

The covariance matrix $C_{yy}$ can then be factorized by applying the singular value decomposition (SVD), i.e.,

(16) $$C_{yy}=V\Lambda V^{T},$$

with V containing the orthonormal eigenvectors and Λ the diagonal matrix containing the eigenvalues, sorted in descending order.

Substituting Equation (16) into Equation (15), we arrive at

(17) $$C_{xx}=W^{T}V\Lambda V^{T}W,$$

with $C_{xx}$ diagonal as soon as the *d* columns of W are taken collinear with *d* columns of V.

In the data-driven modeling approach proposed later, PCA will be used for expressing a time history in a time reduced basis. Thus, instead of describing a time function from its value at different time steps, it will be described by the weight of few functions defined in the whole time interval involved in the time reduced basis.

### 3.2. Code2Vect

In [18] the authors proposed a supervised classification and nonlinear regression procedure that was called *Code2Vect* because of its ability to map data from a representation (non-metric) space into a vector space equipped of a metric.

We assume that points in the origin space consists of *M* arrays composed on D entries, noted by $y_{i}$. Theirs images in the vector space are noted by $x_{i}\in R^{d}$, with in general d≪D. The mapping is described by the d×D matrix W ensuring

(18) $$x_{i}=Wy_{i},\;\;\forall i=1,\cdots ,M,$$

where both, the components of W and the images $x_{i}\in R^{d}$, i=1,⋯,M, must be calculated. Each point $x_{i}$ keeps the label (value of the quantity of interest (QoI)) associated with is origin point $y_{i}$, denoted by $O_{i}$.

We would like placing points $x_{i}$, such that the Euclidian distance with each other point $x_{j}$ scales with their outputs difference, i.e.,

(19) $$\left(Wy_{i}\right)\cdot \left(Wy_{i}\right)=∥x_{i}-x_{j}{∥}^{2}=\left|O_{i}-O_{j}\right|,$$

where the coordinates of one of the points $x_{i}$ can be arbitrarily chosen. Linear mappings are limited and do not allows proceeding in nonlinear settings. Thus, a better choice consists of the nonlinear mapping W(y), expressible from the general polynomial form

(20) $$W\left(y\right)=\sum_{k=1}^{K}W_{k}P_{k}\left(y\right),$$

where $W_{k}$ are d×D matrices and P(y) a polynomial basis.

Equation (19) ensures clustering due to the fact that data with similar output will be placed closer than when the outputs differ significantly. As well as the mapping has been determined, Equation (18) (or its nonlinear counterpart) becomes an efficient nonlinear regression as described in [18].

In the data-driven modeling approach proposed later, *Code2Vect* will be used for defining nonlinear regressions eventually involving heterogeneous data (e.g., the components of the orientation and velocity gradient tensors).

### 3.3. Model Training

In what follows the orientation based on the Jeffery model that ignores interaction will be denoted by the superscript ${•}^{Jeff}$, whereas the orientation evaluated from MD simulations will be denoted by ${•}^{MD}$.

The deviation ${•}^{dev}$ is expected to describe the fiber interactions because the proposed MD model in absence of interactions (with a vanishing Lennard–Jones potential) was able to reproduce very accurately fiber motion as described by Jeffery’s equation (Equation (4)) . The associated rate equation can be written as

(21) $${\dot{a}}^{dev}={\dot{a}}^{MD}-{\dot{a}}^{Jeff}$$

with components

(22) $$\begin{array}{c} {\dot{a}}^{dev}=\left[\begin{array}{ccc} {\dot{a}}_{11}^{dev} & {\dot{a}}_{12}^{dev} & {\dot{a}}_{13}^{dev}\\ {\dot{a}}_{21}^{dev} & {\dot{a}}_{22}^{dev} & {\dot{a}}_{23}^{dev}\\ {\dot{a}}_{31}^{dev} & {\dot{a}}_{32}^{dev} & {\dot{a}}_{33}^{dev} \end{array}\right] \end{array}$$

It is expected that in absence of confinement, size effect, and for a given fiber concentration, ${\dot{a}}^{dev}$ will depend on the flow velocity gradient and the orientation state a itself.

In what follows we consider two scenarios:

- The first is related to the orientation process from a given initial orientation state for different flows characterized by their velocity gradient kept constant during all the flow process;
- The second is more general and looks for expressing the deviation rate as a function of both the applied flow (defined by its changing velocity gradient) and the existing orientation state at a given time instant.

#### 3.3.1. Orientation Evolution in Homogeneous Flows

In this case, the deviation rate at any time is assumed depending on the initial orientation state $a_{0}$ and the applied flow ∇v. Our main purpose is determining accurately $a(t;a_{0},\nabla v)$. For the sake of simplicity and without loss of generality, we assume the same initial orientation, almost isotropic.

The procedure for learning the model consists of the following steps:

- Consider an almost initial isotropic fiber distribution $p_{k}\left(t=0\right)$, k=1,⋯,N, defining the initial orientation tensor $a_{0}\approx I/3$.
- Generate randomly or using a design of experiments (DoE) different flows characterized by the velocity gradients $\nabla v^{j}$, j=1,⋯,M.
- The second invariant of tensors $\nabla v^{j}$, ${\dot{\gamma }}^{j}$, allows defining the maximum simulation time for each flow $t_{max}^{j}$ such that ${\dot{\gamma }}^{j}t_{max}^{j}=\Gamma$, with Γ total strain large enough for reaching an almost steady orientation state.
- Perform the MD integration for all the considered flows from the initial fiber distribution $p_{k}\left(t=0\right)$, leading to $p_{k}^{j}\left(t\right)$, from which the second order orientation tensor $a_{j}^{MD}\left(t\right)$ can be computed at time *t* from
  (23) $$a_{j}^{MD}\left(t\right)=\frac{1}{N}\sum_{k=1}^{N}p_{k}^{j}⊗p_{k}^{j}$$
  that enables calculating its time derivative ${\dot{a}}_{j}^{DM}\left(t\right)$.
- Perform the integration of the Jeffery model from $a_{0}$ for the different flows, to obtain $a_{j}^{Jeff}\left(t\right)$ and its associate orientation rate ${\dot{a}}_{j}^{Jeff}\left(t\right)$.
- The deviation results ${\dot{a}}_{j}^{dev}\left(t\right)={\dot{a}}_{j}^{MD}\left(t\right)-{\dot{a}}_{j}^{Jeff}\left(t\right)$.
- The interval Γ is divided in *D* intervals, and the deviation rate at the associated deformation (the deformation is defined from $\gamma =\dot{\gamma }t$) extracted, i.e., ${\dot{a}}_{j}^{dev}\left(\gamma_{l}\right)$, l=1,⋯,D.
- Thus, each component (m,n) of ${\dot{a}}_{j}^{dev}\left(\gamma_{l}\right)$, ${\dot{a}}_{mn,j}^{dev}\left(\gamma_{l}\right)$, can be expressed as a point in $R^{D}$, $y_{mn,j}^{dev}$, whose *l*—component results ${\dot{a}}_{mn,j}^{dev}\left(\gamma_{l}\right)$.
- Now, the PCA is applied and the first *d* modes retained. For facilitating visualization we will consider in fact the first three modes, i.e., d=3, defining the matrix W. Thus, the image of $y_{mn,j}^{dev}$ is given by
  (24) $$z_{mn,j}^{dev}=Wy_{mn,j}^{dev},$$
  with $z_{mn,j}^{dev}\in R^{d}$.
- Now, *Code2Vec* applies on vectors $G^{j}$ (with $G^{j}$ the vector form of the velocity gradient $\nabla v^{j}$), that are transformed into $g^{j}$ from the nonlinear mapping $W_{mn}\left(G^{j}\right)$, i.e.,
  (25) $$g^{j}=W_{mn}\left(G^{j}\right)G^{j},$$
  where the mapping is constructed by enforcing
  (26) $$∥W_{mn}\left(G^{r}\right)G^{r}-W_{mn}\left(G^{s}\right)G^{s}∥=∥g^{r}-g^{s}∥=∥z_{mn,r}^{dev}-z_{mn,s}^{dev}∥.$$

With orientation model $W_{mn}\left(G\right)$ constructed, as soon as a new orientation trajectory must be evaluated for a given velocity gradient G it suffices to proceed as follows:

- Map G into the vector space according to g=W(G)G;
- Identify the set S containing the closest neighbors $g^{i}$ of g;
- Compute the approximation weights $\omega_{i}$, i=1,⋯,S, depending on the distance between $g^{i}$ and g, for example by using radial basis;
- Interpolate the deviation reduced orientation trajectories according to
  (27) $$z_{mn}^{red}=\sum_{i=1}^{S}\omega_{i}z_{mn,i}^{dev}.$$
- Push up to reconstruct the time evolution according to
  (28) $$y_{mn}^{red}=\sum_{i=1}^{S}\omega_{i}y_{mn,i}^{dev}.$$
  It is important to note that the inverse mapping $R^{d}\to R^{D}$ is not defined, and by this reason we perform the same interpolation that in the reduced space (previous item)

#### 3.3.2. Instantaneous Orientation Behavior

The present case is even simpler. We consider different orientation states, that can be computed by taking some snapshots of the fibers orientation at different time during a complex flow simulation. Then, for each of them, we applied different flows to evaluate the instantaneous orientation rate of change.

The procedure reads:

- Define at t=0, Q initial orientation states $p_{k}^{q}$, k=1,⋯,N & q=1,⋯,Q, using for example a MD simulation in a complex flow.
- Compute the associated orientation tensors $a_{0}^{q}$
  (29) $$a_{0}^{q}=\frac{1}{N}\sum_{k=1}^{N}p_{k}^{q}⊗p_{k}^{q}.$$
- Define J flows defined by their velocity gradient tensor $\nabla v^{j}$, j=1,⋯,J.
- Update the orientation state at time t=Δt from both the MD procedure and from the Jeffery equation, the former proceeding from $p_{k}^{q}$, the last from $a_{0}^{q}$, leading to $a_{j}^{MD,q}\left(t=\Delta t\right)$ (evaluated from $p_{k}^{q,j}\left(t=\Delta t\right)$) and $a_{j}^{Jeff,q}\left(t=\Delta t\right)$ respectively, from which the associated rates can be obtained, respectively ${\dot{a}}_{j}^{MD,q}\left(t=\Delta t\right)$ and ${\dot{a}}_{j}^{Jeff,q}\left(t=\Delta t\right)$, and from both the deviation ${\dot{a}}_{j}^{dev,q}={\dot{a}}_{j}^{MD,q}\left(t=\Delta t\right)-{\dot{a}}_{j}^{Jeff,q}\left(t=\Delta t\right)$.
- Define the M=J×Q data-sets, $y_{qj}$, each one containing the vector representation of $a_{0}^{q}$ and $\nabla v^{j}$, with as quantity of interest, QoI, ${\dot{a}}_{j}^{dev,q}$.
- Now, *Code2Vect* can apply to map each $y_{qj}$ into $z_{mn,qj}$, by constructing for each component (m,n) the model ${\tilde{W}}_{mn}$ (linear or in general nonlinear as previously dicussed)

Now, online, for any a and ∇v, it results y that by applying the mapping leads to $z_{mn}={\tilde{W}}_{mn}y$. As in the previous section, the set of closest neighbors can be defined, and an interpolation performed to obtain ${\dot{a}}^{dev}$, that is used in the time-marching integration procedure according to

(30) $$\dot{a}={\dot{a}}^{Jeff}+{\dot{a}}^{dev},$$

that adds to the standard Jeffery contribution the one extracted from the data related to the deviations.

## 4. Results

In the considered suspension the fiber volume fraction (ϕ) is kept at 0.01 (semi-dilute regime, 0.0025 < ϕ < 0.05 for a fiber aspect ratio of 20). These conditions apply to both procedures previously defined.

### 4.1. Orientation Evolution in Homogeneous Flows

Total 35 cases with different $\nabla v^{j}$ are prepared for this case study (some of them related to pure shear and elongation flows). Out of 35 cases, its aimed to predict the ${\dot{a}}^{dev}$ of the case number 10. The initial orientation state is almost isotropic, whereas the velocity gradient is

(31) $$\begin{array}{c} \nabla v^{10}=\left[\begin{array}{ccc} -1 & 0 & 1\\ 0 & -1 & 0.25\\ 1 & 0.25 & 2 \end{array}\right]. \end{array}$$

**Figure 1:**
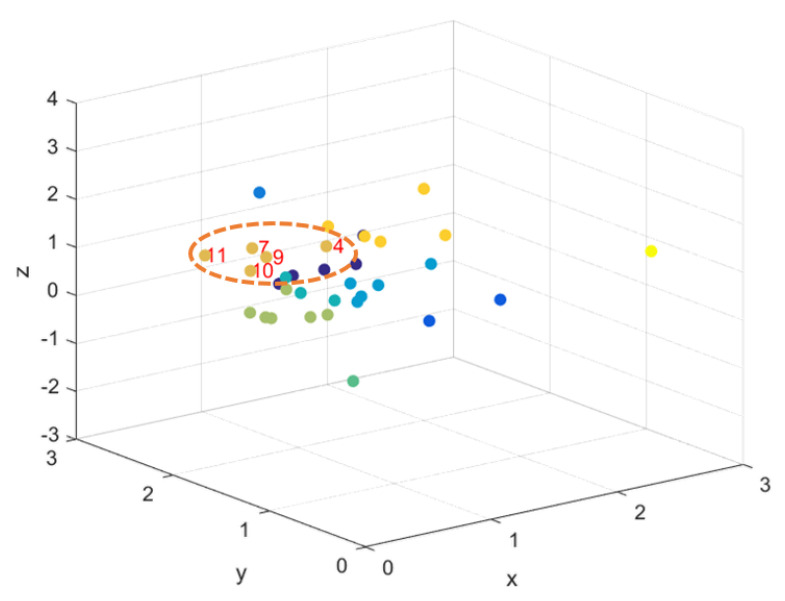
*Code2vect with d=3*. The closest neighbors of the mapped $\nabla v^{10}$ are also indicated.

The prediction for the 10th flow is made according to the procedure described in Section 3.3.1 and is shown in Figure 1. The mapped point representing the 10th flow has as closest neighbors flows 4, 7, 9 and 11.

The velocity gradient $\nabla v^{j}$ for j=4,7,9&11 are:

(32) $$\begin{array}{c} \nabla v_{4}=\left[\begin{array}{ccc} -0.5 & 0.5 & 1\\ 0.5 & -0.5 & -0.75\\ 1 & -0.75 & 1 \end{array}\right],\nabla v_{7}=\left[\begin{array}{ccc} -1 & -0.25 & 0\\ -0.25 & -1 & 1\\ 0 & 1 & 2 \end{array}\right], \end{array}$$

(33) $$\begin{array}{c} \nabla v_{9}=\left[\begin{array}{ccc} -1 & 0 & 0.25\\ 0 & -1 & 0.75\\ 0.25 & 0.75 & 2 \end{array}\right],\nabla v_{11}=\left[\begin{array}{ccc} -1 & 0.5 & -0.5\\ 0.5 & -1 & -0.75\\ -0.5 & -0.75 & 2 \end{array}\right]. \end{array}$$

In what follows, and for the sake of representation clarity, only the component ${•}_{11}$ of the orientation tensor will be discussed.

**Figure 2:**
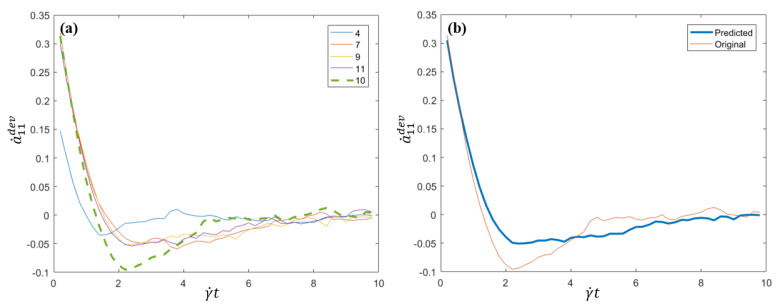
${\dot{a}}_{11}^{dev}$ for flows 4, 7 and 9–11 (**a**) and ${\dot{a}}_{11}^{dev}$ predicted and original data for the tenth flow (**b**).

The corresponding ${\dot{a}}_{11}^{dev}\left(t\right)$ for the five flows *j* = 4, 7, 9, 10, 11 are presented in Figure 2a, whereas the prediction and reference solution (original data) for that flow, j=10, are presented in Figure 2b. The proposed strategy perform quite reasonably by capturing the main behavior. Obviously the finer is the sampling, the more accurate is the prediction.

### 4.2. Instantaneous Orientation Behavior

In the present case we consider two numerical tests reported below.

**Figure 3:**
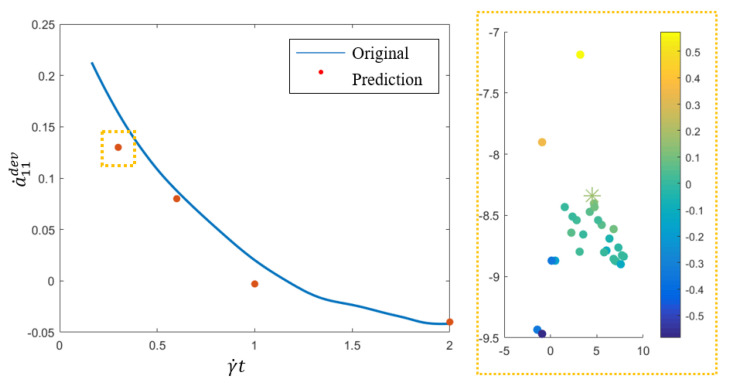
Numerical results.

**Figure 4:**
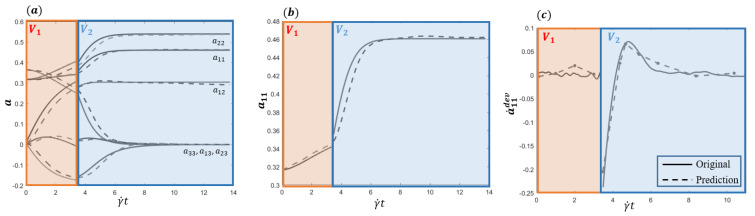
Time evolution of the data-based enriched Jeffery solution (dotted line) with respect to the molecular dynamics (MD) solution (continuous) (**a**,**b**) and ${\dot{a}}_{11}^{dev}$ prediction versus the reference data (**c**).

- In this first test, we perform a MD simulation for extracting different orientation states when considering an almost isotropic initial orientation state a the flow characterized by
  (34) $$\begin{array}{c} \nabla v=\left[\begin{array}{ccc} -0.75 & 0.5 & 0\\ -0.5 & -0.75 & 0\\ 0 & 0 & 1.5 \end{array}\right] \end{array}$$
  The extracted Q=4 intermediate orientation states are defined in Table 1.
  For these four orientation states a total of 38 samples at different ∇v are prepared.
  As discussed in Section 3.3.2 *Code2Vect* applies on the *M* data $\nabla v^{j}$ and $a_{0}^{q}$ whose results are presented in Figure 3, where original data and predictions of ${\dot{a}}_{11}^{dev}$ up to $\dot{\gamma }t=2$ is depicted (left). Prediction at the evaluation point (star) along with 37 samples (filled circle) is depicted on the right image of that figure, where color scales with the value of ${\dot{a}}_{11}^{dev}$ at $\dot{\gamma }t=0.3$. The predicted data shows a good agreement with the raw data validating the proposed methodology.
- The last numerical test addresses a more complex scenario where the velocity gradient changes within the simulation-time window.
  The prediction for ${\dot{a}}^{dev}$ is made when fibers (almost isotropically distributed at the initial time) are placed under two velocity gradient fields, $\nabla v_{1}$ (shear) and $\nabla v_{2}$ (elongation), consecutively, the former applying during the first 3.3 s and the last the subsequent 10 s.
  The velocity gradient ∇v for these flow fields is
  (35) $$\begin{array}{c} \nabla v_{1}=\left[\begin{array}{ccc} 0 & 0.75 & 0.25\\ 0.75 & 0 & -0.5\\ 0.25 & -0.5 & 0 \end{array}\right],\nabla v_{2}=\left[\begin{array}{ccc} 1 & 0 & 0\\ 0 & 1 & 0\\ 0 & 0 & -2 \end{array}\right]. \end{array}$$
  Data-driven enriched Jeffery solution and the one provided by MD simulation are compared over the entire time period in Figure 4a,b, whereas Figure 4c depicts the deviation prediction (following the procedure described in Section 3.3.2) versus the reference one provided directly by the data. As it can be noticed, the numerical values of the prediction when compared with the reference data, both reported in Table 2, exhibit an excellent accuracy.

## 5. Conclusions and Prospects

This work shows the opportunity of hybrid modeling methodologies, combining a model based on physics complemented with a data-driven enrichment to account for the gap between the model predictions and the collected data. In this work the synthetic data was used from a high-fidelity fine-scale simulation based on molecular dynamics, a simulation which took fiber–fiber interactions into account.

The data-driven model was constructed by using advanced machine learning techniques able to operate at the low-data limit, in particular the PCA and the so-called *Code2Vect*.

The proposed methodology was verified with numerical experiments that show the efficiency of our hybrid approach and the techniques employed for constructing the data-driven model.

Currently injection molding simulations use a constitutive model in which they have to provide an interaction parameter which is the same everywhere and is determined from experiments conducted under simple shear. It has been shown that the flow field is very different around corners, inserts, ribs and undercuts in complex injection molded parts. It is expected that the constitutive interaction parameter locally will be different but there is no model or methodology to determine this. Our approach should allow for more accurate fiber orientation prediction in such situations. The extension of the present work to efficient processing simulations constitute a work in progress.

## Figures and Tables

**Table 1:** $a_{0}$ at $\dot{\gamma }t$ = 0.3, 0.6, 1 and 2.

$\dot{\gamma }t$	$a_{11}$	$a_{22}$	$a_{33}$	$a_{12}$	$a_{13}$	$a_{23}$
0.3	0.32	0.30	0.36	−0.01	−0.01	0.01
0.6	0.28	0.27	0.43	−0.03	−0.01	0.01
1	0.22	0.21	0.56	−0.05	−0.01	0.01
2	0.14	0.13	0.71	−0.06	−0.01	0.01

**Table 2:** Raw data and predicted data from Figure 4 at different time instants.

$\dot{\gamma }t$	Raw Data: ${\dot{a}}_{11}^{\mathrm{dev}}$	Predicted Data: ${\dot{a}}_{11}^{\mathrm{dev}}$
0.1	0.004	−0.03
1	0.0019	0.0034
2	0.003	0.02
3	−0.001	0.002
3.4	−0.249	−0.205
4.3	0.038	0.025
4.6	0.067	0.066
5.3	0.048	0.044
6.3	0.008	0.025
8.3	0.006	−0.002
10.3	−0.002	−0.004
